# Bilateral Asynchronous Necrotizing Sialometaplasia of the Buccal Mucosa: A Case Report and Literature Review

**DOI:** 10.7759/cureus.24136

**Published:** 2022-04-14

**Authors:** Elle Nuttall, Daniel Wehrmann

**Affiliations:** 1 Otolaryngology-Head and Neck Surgery, Creighton University, School of Medicine, Omaha, USA; 2 Otolaryngology-Head and Neck Surgery, Children's Hospital and Medical Center, Omaha, USA

**Keywords:** differential diagnosis, oral surgeries, oral pathologies, oral mucosal lesions, oral diseases, buccal mucosa, necrotizing sialometaplasia

## Abstract

Necrotizing sialometaplasia is a rare, benign disease that affects any area containing minor salivary glands. This entity presents a diagnostic difficulty due to its resemblance to malignancy. A unique case of bilateral asynchronous necrotizing sialometaplasia with superinfection secondary to trauma is described in this study. A 19-year-old female presented with bilateral ulcerative lesions in her buccal mucosa and facial swelling. The two lesions appeared several weeks apart. The diagnostic workup excluded rheumatologic, malignant, and infectious etiologies. The patient was treated with antibiotics and steroids with subsequent resolution of symptoms. Given the exclusion of other etiologies, the patient was diagnosed with bilateral asynchronous necrotizing sialometaplasia with superinfection. This case demonstrates the importance of considering necrotizing sialometaplasia as a diagnosis in all patients with oral ulcerative lesions.

## Introduction

Necrotizing sialometaplasia (NSM) is a rare, self-limiting disease that may arise from any area containing salivary gland tissue [1,2]. Although NSM involves the hard palate in 80% of cases, lesions may arise in the nasal and paranasal sinuses, parotid gland, sublingual gland, submandibular gland, laryngotracheal complex, and oral cavity subsites [1-3]. NMS presents a unique diagnostic dilemma as it may mimic squamous cell carcinoma or mucoepidermoid carcinoma [3-6].

Management of NSM primarily includes surveillance and reassurance, as these lesions typically resolve spontaneously in four to 10 weeks [3,7]. We present a case of asynchronous bilateral necrotizing sialometaplasia with bacterial superinfection.

## Case presentation

A 19-year-old female was evaluated in the emergency department for complaints of increasing left-sided cheek pain and swelling. The patient had a five-month history of ulceration of the buccal mucosa that was thought to be due to an impacted wisdom tooth and having braces. She noted two prior episodes of left-sided facial swelling in the months preceding that resolved with courses of amoxicillin. The patient was a chronic marijuana user and smoked twice daily.

On examination, the patient had left-sided facial swelling with overlying erythema. An oral examination revealed a 4 cm area of irregular mucosa with underlying nodularity and 2 cm ulceration with fibrinous debris just lateral to the left retromolar trigone and several areas of early ulceration. No cranial nerve deficits were noted.

Laboratory workup indicated leukocytosis with WBC 14.4 K/uL on complete blood count and an unremarkable complete metabolic panel. Culture of the lesion grew mixed anaerobic flora. CT scan of the face demonstrated subcutaneous fat stranding with soft tissue swelling and an enlarged left submandibular glandwith reactive left 2A lymph nodes. The biopsy of the ulcerative lesion noted squamous mucosa with underlying superficial and deep acute and chronic inflammation. The differential diagnosis included malignant, rheumatologic, and infectious etiologies. The patient was prescribed 14 days of amoxicillin/clavulanic acid and noted resolution of the pain and swelling. Subsequently, the presumed diagnosis of necrotizing sialometaplasia with superinfection was made.

Two weeks after the initial emergency department visit, the patient was again evaluated in the emergency department due to pain and swelling of her contralateral cheek. The patient had four molars extracted four days prior to this presentation. This extraction was complicated by malocclusion, which resulted in the patient repeatedly biting down on her buccal mucosa. She was given the diagnosis of presumed right buccal mucosa cellulitis. The patient was sent home with amoxicillin/clavulanic acid, triamcinolone paste, and a methylprednisolone course.

Two weeks after this second emergency department visit, the patient again presented to the emergency department with a several-week history of pain and swelling in her right cheek. She stated that the pain and swelling had dramatically increased over the previous 24-hour period, prompting her to seek emergency care. She had been taking her medications as prescribed. Associated symptoms included right-sided blurry vision, right-sided facial twitching, right-sided facial droop, right ear pain, submandibular pain, dry mouth, dizziness, weakness, drowsiness, nausea, and vomiting. She also reported an unintentional 15-pound weight loss over the last four months.

**Figure 1:**
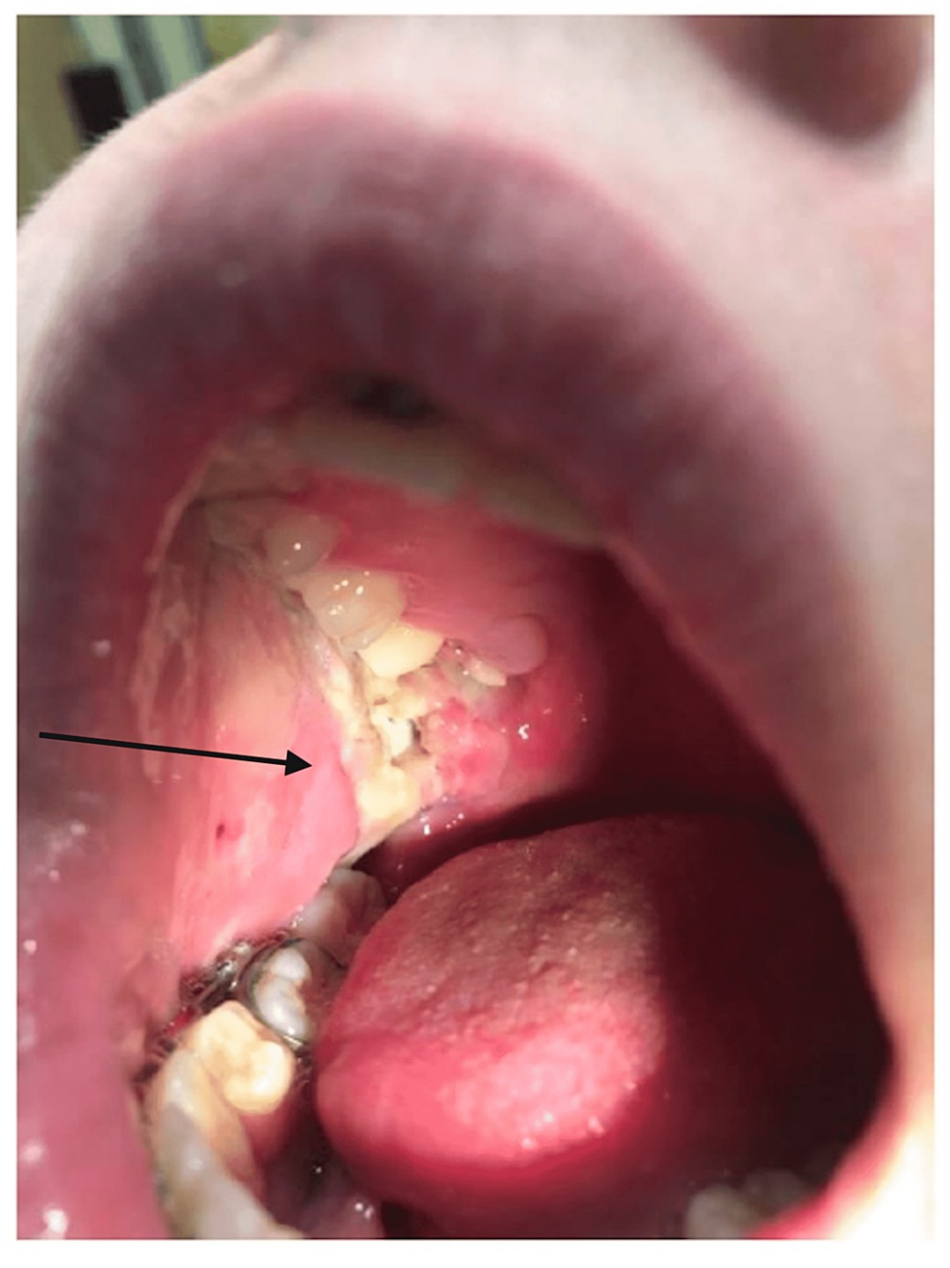
Ulceration of right buccal mucosa and retromolar trigone.

**Figure 2:**
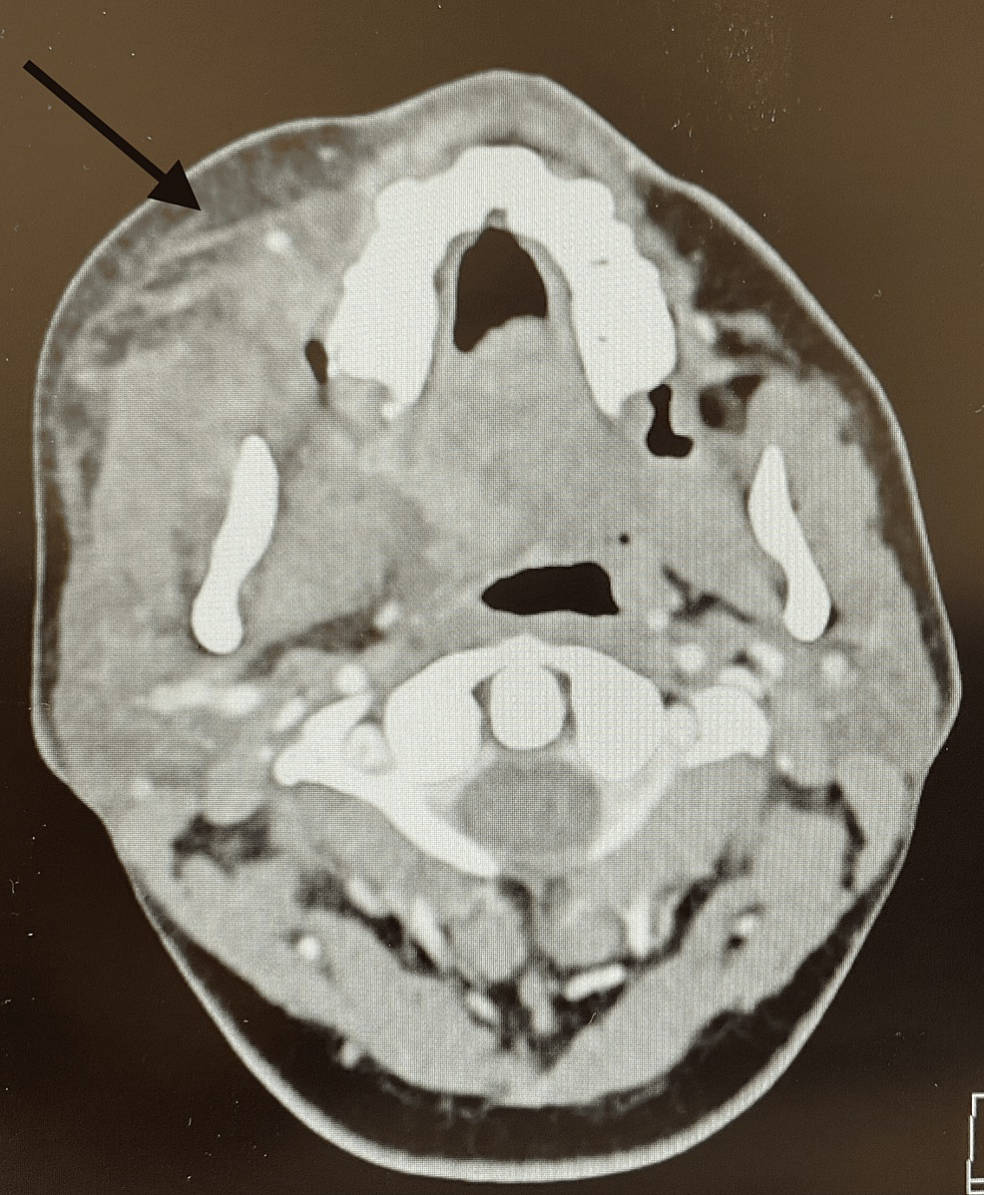
CT demonstrating severe edema of the pterygoid and masseter muscles with fat stranding.

Physical examination revealed ulceration of the mucosa in right retromolar trigone, right facial soft tissue swelling, and superficial ulceration of the right cheek (Figure 1). Right cheek crepitus and right-sided cervical lymphadenopathy were appreciated on palpation. There was no trismus on examination. Tympanic membranes and external ears appeared within normal limits. The patient also had superficial ulceration on her right cheek due to ice pack usage. No cranial nerve deficits on examination. The patient was subsequently admitted for pain control and an infectious, malignant, and rheumatologic workup. The differential diagnosis included bacterial or fungal infection, necrotizing sialometaplasia with superinfection, malignancy, abscess, and other possible infectious etiologies included diabetes mellitus, tuberculosis (TB), HIV, and immunodeficient status. Laboratory workup was significant for leukocytosis of 21.5 K/uL with neutrophilic predominance with polymorphonuclear leukocytes (PMN) 84.9%, thrombocytosis with a platelet count of 474 K/uL, and an elevated C-reactive protein (CRP) of 16 mg/dL and erythrocyte sedimentation rate (ESR) of 87 mm/h. A complete metabolic panel was unremarkable including blood glucose was within normal limits. C4, C3, IgA, IgM, IgG, and tissue transglutaminase antibody IgA were within normal limits. Infectious workup was negative for herpes simplex virus, HIV, and hepatitis C. Culture of the lesion grew *Streptococcus anginosus* and mixed respiratory flora. Fungal cultures were negative. Two separate biopsies of the lesion noted nonspecific inflammation with ulcerated granulation tissue without characteristics of malignancy. A CT scan of the neck demonstratedright maxillary hemorrhage, severe swelling of the pterygoid and masseter muscles with fat stranding, right-sided cervical lymphadenopathy, and near-complete opacification of the right maxillary sinus (Figure 2). An MRI of the face was subsequently obtained for more detailed imaging to rule out bony involvement. MRI did not reveal any evidence of osseous involvement.

The patient was prescribed high-dose intravenous steroids and intravenous ampicillin/sulbactam. With improvement of the edema and pain, the steroids were subsequently discontinued after two days. The patient was discharged on acetaminophen and ibuprofen. She was continued on a course of amoxicillin/clavulanic acid and clindamycin when seen on outpatient a week later. On outpatient follow-up two weeks after discharge, the swelling was continuing to resolve albeit more slowly than anticipated. Buccal ulceration was significantly improved, but still present. Given that other etiologies of mucosal ulceration were ruled out, the diagnosis of necrotizing sialometaplasia with superinfection was considered.

## Discussion

This case presented a unique diagnostic challenge given the asynchronous nature of the contralateral lesion and superinfection creating an inflammatory picture. Pathophysiology of necrotizing sialometaplasia (NSM) is poorly understood, but the predominant theory suggests that this lesion is due to ischemia of the salivary gland lobules [1-4]. Traumatic injuries, such as local anesthetic, vasculopathies, chronic vomiting seen in bulimia nervosa, cocaine use, smoking, local radiotherapy, and space-occupying lesions, have been considered as potential predisposing factors to ischemia [2-6]. Similar to the etiology of erythema multiforme or trigeminal sensory neuropathy, upper respiratory infection has been associated with NSM likely due to immune complexes causing ischemia [5]. Trauma is the most likely etiology of this patient’s case of bilateral asynchronous NSM given her longstanding history of orthodontic procedures complicated by chronic buccal ulcerations. The patient’s daily marijuana use may have also been a contributing factor in the development of these lesions.

NSM is only cited in 0.03% of all biopsied oral lesions; however, this value may be underestimated as the condition is poorly recognized [6]. NSM occurs in patients aged 17-80 years with a male predominance of 2:1. The majority of patients diagnosed with NSM are over the age of 40 years [3,4]. NSM typically presents as a well-demarcated, deep ulcer ranging from 0.5-5 cm with surrounding erythema [2]. While two-thirds of lesions are unilateral, midline, bilateral synchronous, and metachronous lesions can occur [2,3]. Patients with NSM will often complain of a painful lesion with antecedent fever, malaise, and swelling [3,5]. There have also been reports of localized paresthesia and referral of pain to the ear and pharynx [5]. This patient’s initial presentation was consistent with previously reported cases of NSM. However, this presentation was unique in that she developed contralateral lesions located in the buccal mucosa near the retromolar trigone two weeks after the initial presentation. With the second lesion, she reported localized paresthesia in the V2 trigeminal distribution, subjective facial droop, and referred right ear pain with normal-appearing external ears and tympanic membranes. Haen et al. reported a case of NSM involving the parotid gland that resulted in facial nerve paralysis, an uncommon occurrence [8]. Our patient's subjective facial droop may have been due to edema limiting facial movement.

**Figure 3:**
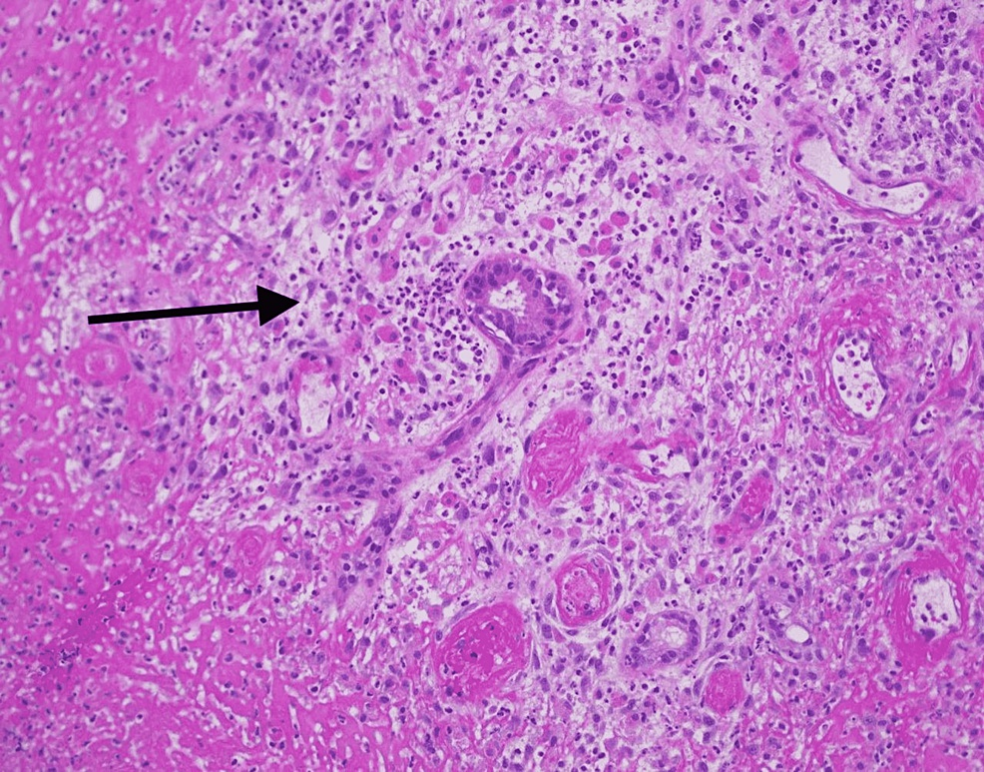
Markedly inflamed and ulcerated granulation tissue on hematoxylin and eosin stain from biopsy during inpatient admission.

NSM has broad differential diagnosis including infectious, immunologic, malignant, and metabolic etiologies [3,9,10]. Diagnosis of NSM is often based on clinical presentation correlated with histopathology [2,3]. It is important when obtaining a histopathology sample of these lesions to biopsy the viable end of the ulcer, thus avoiding sampling only necrotic material [1]. This technique was utilized in this case. NSM has five key histopathologic features. These include pseudoepitheliomatous hyperplasia, squamous metaplasia of ducts and acini, preservation of lobular architecture, lobular infarction, and inflammation secondary to extravasation of mucin [2-4,6,9]. Carlson suggests that preservation of the lobular architecture is a key characteristic [4]. Histopathology may vary depending on time of diagnosis. Early cases present with coagulative necrosis while late cases often present with squamous metaplasia of ducts with reactive fibrosis [5,7,9]. A case report by Senapati et al. demonstrated two cases of NSM that exhibited histopathologic evidence of a vascular ischemic origin [1]. These cases presented with ischemic necrosis of the salivary gland acini with replacement by squamous metaplasia [1]. Morphology resembling NSM has been seen in the periphery of salivary glands involved with cancer, which raises suspicion that this may be a precancerous lesion [6]. Given the nonspecific inflammation seen on histopathology in this case, malignancy was ruled out and the diagnosis of necrotizing sialometaplasia was considered (Figure 3). This patient’s laboratory workup was also negative for rheumatologic and infectious etiologies.

Primary management of these lesions is surveillance and reassurance, as these lesions typically resolve spontaneously in four to 10 weeks without treatment beyond pain control [3,5,7]. It is important to consider NSM in the differential in patients with a similar presentation, as misdiagnosis can result in unnecessary and high-risk treatments including aggressive surgery and steroid therapy [3,5]. Due to the self-limiting nature of these lesions, no treatment is necessary other than pain management [2]. Given the concern for a rheumatologic etiology, this patient was started on high-dose intravenous steroids. In a case report of bilateral palatine NSM by Keogh et al., a trial of intralesional steroid injection was done on one lesion with the other serving as a control [5]. There was no significant improvement found in the lesion that received steroid injection when compared to the control lesion [5]. This suggests that steroids may not be an effective treatment for NSM. The steroids were subsequently discontinued as the patient’s swelling began to improve. This case was also treated with antibiotics given the suspected superinfection in the lesions. Given the difficulty of diagnosis and risk of aggressive management including possible surgery in NSM, it is an important diagnosis to consider in patients that present with oral lesions.

## Conclusions

Given the diagnostic difficulty and risk for aggressive management strategies, necrotizing sialometaplasia is an important diagnostic entity to consider in patients with oral ulcerative lesions. Reassurance and observation are the primary management strategy, as these lesions spontaneously resolve over time. This case demonstrates one of the many unique presentations of necrotizing sialometaplasia. The asynchronous nature of the lesions and superinfection also complicated this patient’s presentation and management. This case necessitated a thorough diagnostic workup and further enforces the significance of considering this diagnosis.
